# A Retrospective Analysis of Ambiguous Spitz Tumors Using Next-Generation Sequencing

**DOI:** 10.3390/cancers17071227

**Published:** 2025-04-04

**Authors:** Mario Teufer, Martin Theiler, Joana Lanz, Lisa Weibel, Ulrich Wagner, Mitchell P. Levesque, Jivko Kamarachev, Reinhard Dummer, Egle Ramelyte

**Affiliations:** 1 Department of Dermatology, University Hospital Zurich, 8091 Zurich, Switzerland; mario.teufer@usz.ch (M.T.); mitchell.levesque@usz.ch (M.P.L.); jivko.kamarachev@usz.ch (J.K.); reinhard.dummer@usz.ch (R.D.); 2 Faculty of Medicine, University of Zurich, 8006 Zurich, Switzerland; martin.theiler@kispi.uzh.ch (M.T.); joana.lanz@kispi.uzh.ch (J.L.); lisa.weibel@kispi.uzh.ch (L.W.); ulrich.wagner@usz.ch (U.W.); 3 Pediatric Skin Center, Department of Dermatology, University Children’s Hospital Zurich, 8032 Zurich, Switzerland; 4 Department of Dermatology and Allergology, Kantonsspital St. Gallen, 9007 St. Gallen, Switzerland; 5 Department of Pathology and Molecular Pathology, University Hospital Zurich, 8091 Zurich, Switzerland

**Keywords:** Spitz tumors, next-generation sequencing, melanoma-specific panel, atypical Spitz tumors, immunohistochemistry

## Abstract

**Background:** Spitz tumors (STs) are a diverse group of melanocytic lesions that range from benign to malignant. STs pose significant classification challenges due to overlapping histological and immunohistochemical (IHC) features among the STs with different malignant potential. This study aimed to assess the diagnostic value of a melanoma-specific next-generation sequencing (NGS) panel (MelArray) combined with IHC analysis to improve the assessment of diagnostically challenging ST cases. **Methods:** Patients with STs and available MelArray results were included in this retrospective analysis. Molecular analysis (genetic alterations, tumor mutational burden (TMB), and copy number variations (CNV)), clinical data (demographics and clinical course), and IHC data (scores for markers such as p16, Ki-67, HMB45, PRAME, and Melan A) were evaluated in conjunction and correlated with patient outcomes. **Results:** Atypical Spitz tumors (ASTs, *n* = 20) predominantly exhibited heterozygous deletions in melanoma-relevant genes, but these were not accompanied by the multiple damaging mutations commonly associated with melanoma. IHC scores were higher in ASTs compared to Spitz nevi (SN, *n* = 3), suggesting an intermediate biologic potential. SN exhibited minimal genetic alterations and low IHC scores, reflecting a benign profile. Genetic analysis of the Spitz melanoma (SM, *n* = 1) revealed a distinct molecular profile with damaging mutations affecting the key regulatory pathways involved in tumor progression, along with a high TMB, and an IHC score comparable to ASTs. During a median follow-up of 36 months (range: 6–48 months, *n* = 23), no recurrences, distant metastases, or tumor-related deaths were observed. **Conclusions:** The integration of NGS analysis with the MelArray panel, histology, and immunohistochemistry, enhances the diagnostic accuracy of challenging STs by identifying the genetic alterations linked to malignancy risk. This aids in the detection of high-risk lesions that need a more detailed work-up and more stringent follow-up, and those that will follow a benign course. Larger studies are needed to validate the clinical utility and broader applicability.

## Simple Summary

Spitz tumors are a group of melanocytic skin lesions that are difficult to evaluate due to the overlapping characteristics between benign and malignant tumors. This study evaluated 24 ambiguous Spitz tumors, where a melanoma-specific next-generation sequencing panel (MelArray) was used in combination with immunohistochemical analysis to enhance the diagnostic accuracy. The results revealed that benign Spitz nevi showed minimal genetic alterations, whereas atypical Spitz tumors predominantly exhibited heterozygous deletions in melanoma-associated genes but lacked the multiple damaging mutations typically observed in melanoma. One Spitz melanoma was analyzed and displayed damaging mutations in key regulatory pathways and a high tumor mutational burden, both of which are commonly associated with melanoma. Combining molecular insights from a melanoma-specific NGS panel with immunohistochemical analysis provides a valuable approach for a more precise diagnosis, personalized risk assessment, and thus the improved management of ambiguous Spitz lesions.

## 1. Introduction

STs are a heterogeneous group of melanocytic neoplasms that are characterized by their spindle and/or epithelioid cell morphology and can exhibit varying biological potential [1]. Pathologist Sophie Spitz was the first to describe these tumors in 1948, initially referring to them as melanomas of childhood [2]. These specific morphologically distinct entities of melanocytic lesions were later renamed as STs [2]. STs generally show a rapid growth phase, and about 70% of the cases are diagnosed within the first two decades of life, although presentation in adulthood is possible [3]. While most melanocytic tumors can be classified unambiguously as benign nevi or melanomas, these neoplasms pose a particular challenge due to their often ambiguous morphological, histological, and IHC features, which complicate accurate classification, patient management, and the treatment strategy [4,5,6]. Some STs can be readily classified histopathologically as either benign SN or malignant SMs. Clinical and histological features such as symmetry, cell morphology, mitotic activity, depth of invasion, nuclear pleomorphism, and specific dermoscopic patterns may help to differentiate SN from SMs [6,7,8,9]. However, there are also STs that exhibit heterogeneous histopathological and IHC characteristics indicative of both benign and malignant processes [4,5,10,11]. From a morphological perspective, many features that are used to identify melanocytic neoplasms as malignant, such as pagetoid spread, dermal mitotic activity, and nuclear atypia, can also be present in certain subgroups of STs [6]. Tumors that display benign or intermediate clinical behavior despite their “malignant” morphology are classified as ASTs or Spitz tumors with an uncertain malignant potential (STUMP) [6,12]. Recently, the term, Spitz melanocytoma, was introduced in the 2022 WHO classification to describe lesions with intermediate biological potential, previously referred to as ASTs or an STUMP [13]. While we acknowledge this updated terminology, we continue to use the established term, AST, for consistency with the current literature and diagnostic practice.

ASTs, for example, frequently metastasize into regional lymph nodes but do not spread hematogenously [14,15]. This intermediate biologic potential, combined with the difficult histopathological classification, complicates the traditional classification of melanocytic tumors as either completely benign or malignant [7]. Lallas et al. [15] showed in a systematic review that the prognosis for patients with ASTs and local lymph node metastases is generally favorable, and therefore a sentinel lymph node biopsy (SLNB) does not provide prognostic benefit. In contrast, an SM has the potential for extranodal and hematogenous metastasis and are associated with a potentially fatal course [16]. Thus, ASTs represent a difficult and controversially discussed subgroup of STs.

Recent advancements in molecular diagnostics, especially NGS, have provided valuable insights into the genetic drivers of STs and can sometimes also offer prognostic information. Most STs are driven by translocation-induced fusions, present in approximately 50% of cases [8]. These fusions typically involve genes encoding receptor tyrosine kinases, such as ALK, ROS1, NTRK1-3, RET, and MET, or serine-threonine kinases like BRAF, MAP3K3, and ERBB4 [17,18,19]. In contrast, only a minority, around 20%, harbor activating point mutations, most commonly in HRAS [8,20,21,22]. Melanocytic lesions with spitzoid histological features that harbor activating BRAF (mostly BRAFV600E), NRAS, or inactivating of NF1 mutation, which often act as drivers in conventional nevi or melanomas, are referred to as “Spitzoid melanomas” and, according to the latest WHO classification, are no longer considered to be part of the “true ST family” [23]. The WHO now classifies STs as having both a characteristic spitzoid morphology and a defining genomic fusion or HRAS mutation [23]. In a study by Quan et al. [24], 81% of STs were found to harbor kinase fusions and/or truncations, acting as an oncogenic driver in these lesions. However, a subset of lesions remains without identifiable genetic alterations, complicating the classification of the remaining STs [4]. Furthermore, there are no histopathological criteria or molecular markers that are sensitive and specific enough to reliably distinguish ASTs from SMs [8,12,14,25]. To date, the only clear indicator of malignancy in STs is the presence of distant metastasis or death [26].

The aim of this retrospective analysis was to assess the diagnostic value of a customized melanoma-specific NGS panel (MelArray) of 190 genes in classifying STs, with a particular focus on those with uncertain malignant potential (AST/STUMP). By identifying specific genetic markers, we seek to differentiate between benign SN, ASTs, and malignant SMs, and to improve the assessment of the malignant potential of these lesions. This project will correlate genetic findings with IHC and clinical data to enhance the diagnostic accuracy and improve the patient management in STs.

## 2. Materials and Methods

### 2.1. Study Design and Patient Selection

**Figure 1:**
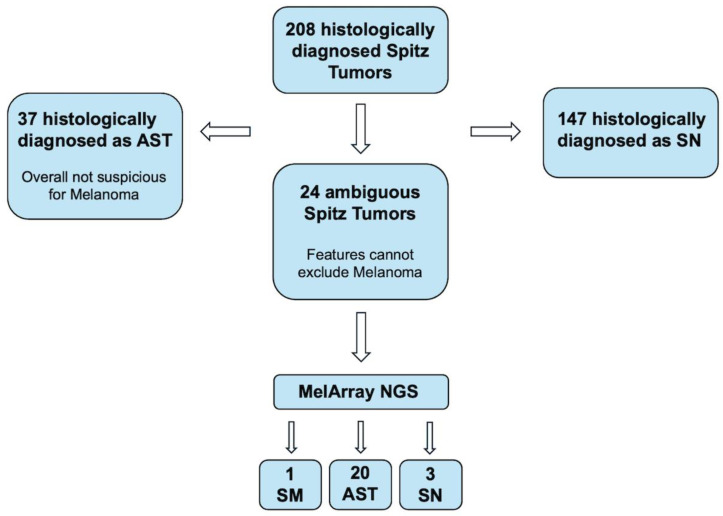
Representative flowchart illustrating the identification process of Spitz tumor patients. Abbreviations: Spitz nevi (SN), atypical Spitz tumor (AST), Spitz melanoma (SM).

This study was designed as a retrospective analysis. Patients were included if they were diagnosed with STs between January 2018 and July 2024 at the University Hospital of Zurich (USZ) or University Children’s Hospital Zurich, and had available NGS data from the MelArray panel, histological and IHC analyses, as well as clinical and demographic data. Patients were identified using the DermaPro (ifms GmbH, Saarbrücken, Germany) database, employing keywords such as “Spitz tumor”, “Spitz nevus”, “Atypical Spitz tumor”, “Spitz melanoma”, and “Spitz tumor of indeterminate/unknown malignant potential” (Figure 1). Among 208 identified cases, 147 were histologically classified as SN and 37 as AST. Molecular testing with the melanoma-specific NGS panel MelArray (Oncobit, Schlieren, Switzerland) was performed in 24 ambiguous cases. In a subset of these cases (*n* = 12), additional gene fusion analysis was conducted using NGS-based assays: Oncomine Assay (Thermo Fisher Scientific, Waltham, MA, USA, 10/12 cases), FoundationOne Heme (Foundation Medicine, Cambridge, MA, USA, 1/12 case), or FusionPlex Salivary Gland Panel (ArcherDx, Boulder, CO, USA, 1/12 case). The final diagnosis was made by integrating the clinical presentation, histological features, and molecular findings. The genetic criteria used that favored the diagnosis of SN, ASTs, or SMs were as follows: SN were characterized by the absence of mutations in melanoma-relevant genes; ASTs presented with isolated mutations or heterozygous deletions in specific melanoma-associated genes; SMs exhibited alterations in two or more key regulatory mechanisms involved in melanoma pathogenesis. Follow-up data were obtained through a review of patient records at the USZ and the University Children’s Hospital Zurich, or by contacting their treating physicians.

### 2.2. Immunohistochemical Analysis

IHC evaluations were conducted with varying frequency for each marker. A scoring system was used to assess p16, Ki-67, and HMB45 individually, based on previously reported methodologies for conventional melanoma and STs [25,27]. PRAME and Melan A were additionally evaluated using a scoring approach. Each patient’s scores were compiled into a total IHC score (range 0–13) (Table 1).

### 2.3. Molecular Testing

The MelArray panel is a targeted NGS assay designed to analyze alterations in 190 melanoma-relevant genes (Supplementary Table S1). This panel enables the detection of point mutations, insertions, deletions, and amplifications, and evaluates TMB and CNV. It also identifies hotspot mutations, which have been linked to a potentially malignant course in STs in previous studies [14,26]. NGS analysis using the MelArray panel followed a modified version of the previously described protocol used by Fröhlich et al. [28]. DNA was extracted using the Maxwell 16 System Purification Kit (Promega, Madison, WI, USA) and quantified using Qubit (Thermo Fisher Scientific, Waltham, MA, USA). Library preparation was performed using the KAPA HyperPrep Plus Kit (Roche Sequencing Solutions, Pleasanton, CA, USA). Hybrid capture was conducted using the SeqCap EZ Choice Kit (Roche, Nimblegen, Madison, WI, USA), following Nimblegen protocols that were optimized during in-house validation. Sequencing was performed on an Illumina NextSeq500/550 (San Diego, CA, USA) platform using the NextSeq High Output Kit. Data analysis was carried out using a pipeline specifically programmed for the MelArray panel, aligned to the GRCh37 genome version.

### 2.4. Data Analysis

Data analysis was performed using R software (version 4.3.3, R Core Team, 2024) and R Studio (version 2024.09.0+375) for data visualization. Prism software (version 9.10) was used for statistical analysis and graph generation. Data analysis was descriptive and without formal statistical testing.

## 3. Results

### 3.1. Study Cohort

Twenty-four patients were diagnosed with ambiguous STs requiring NGS for further diagnostics. Among them, three patients (12.5%) were diagnosed with SN, twenty patients (83.3%) were diagnosed with ASTs, and one patient (4.2%) was diagnosed with an SM. Fourteen patients (58%) were female, and ten patients (42%) were male. The age ranged from 2 to 46 years old, with a median age of 15 years old. The most common tumor locations were the lower extremities in 10 patients (42%) and the head in 7 patients (29%). Re-excision was performed in 18 patients (75%), with safety margins of 0.5 cm in 12 patients (67%), 1 cm in 5 patients (28%), and 2 cm in 1 patient (5%). The size of the safety margins depended on multiple factors, including the final diagnosis, the margin status from the initial biopsy, and the tumor location. SNLB was performed in three patients (12.5%) and was positive in one patient (33%). No local recurrences, distant metastases, or tumor-related deaths occurred during the median follow up of 36 months (range: 6–48 months, *n* = 23), but one patient diagnosed with ASTs was lost to follow-up (Table 2).

### 3.2. Immunohistochemistry

**Figure 2:**
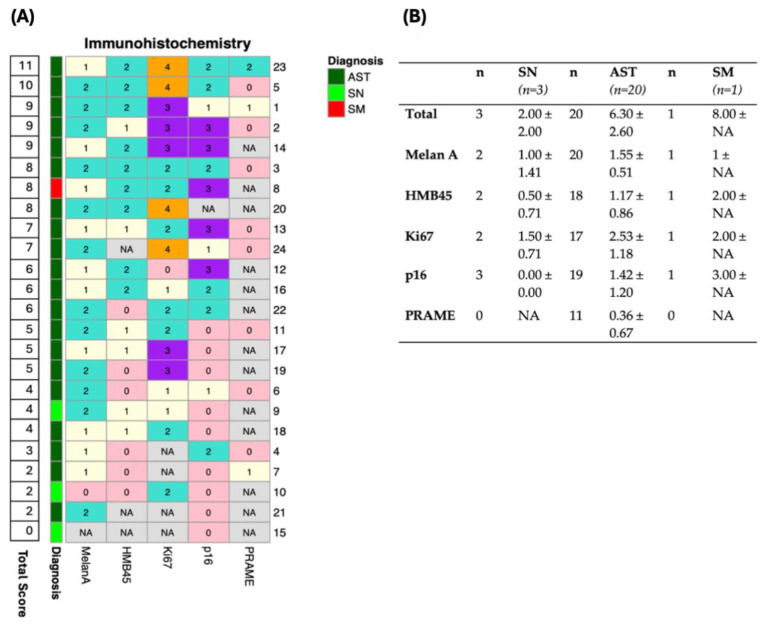
Immunohistochemical staining results across Spitz nevi, atypical Spitz tumors, and Spitz melanoma. (**A**) Heatmap of immunohistochemical staining results for five markers (Melan A, HMB45, Ki67, p16, PRAME) across patient samples. Patients are ranked in descending order by total immunohistochemistry score, and diagnoses are color-coded as atypical Spitz tumor (AST, dark green), Spitz nevi (SN, light green), and Spitz melanoma (SM, red). Marker expression is represented by color intensity, with missing values indicated as “NA”. (**B**) Summary table of mean staining scores ± standard deviation for each marker across the three diagnostic groups: SN, AST, and, SM, as well as for the total cohort. The number of samples (*n*) analyzed for each marker is shown.

IHC staining was applied with varying frequencies across the cohort (Figure 2). The mean IHC score was 2.00 ± 2.00 for SN, 6.30 ± 2.60 for ASTs, and 8.00 for SMs. However, PRAME staining was not applied in the SN and SM groups, limiting direct comparability.

**Figure 3:**
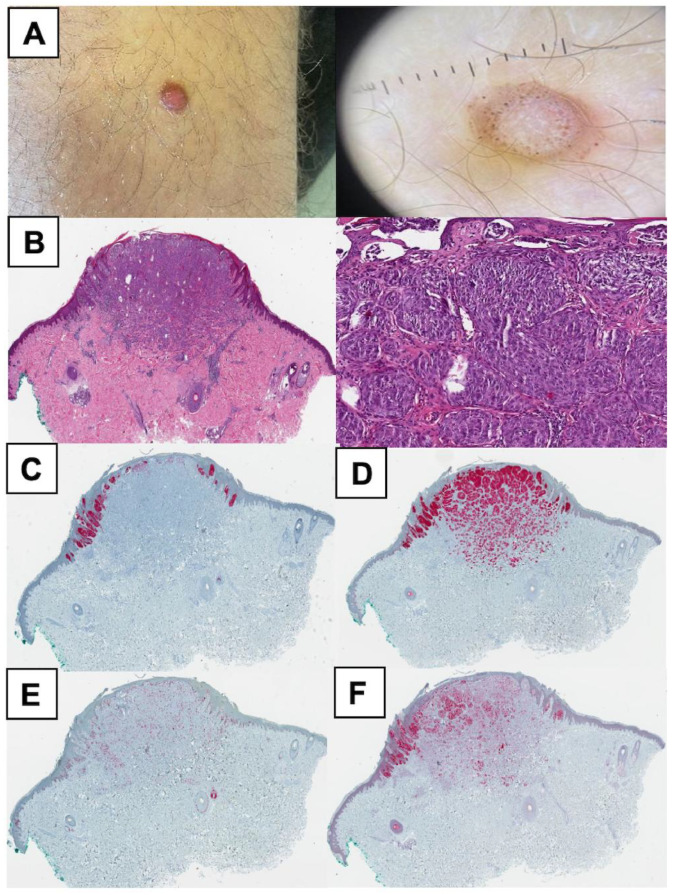
Overview of clinical, histological, and immunohistochemical findings in an atypical Spitz tumor patient (Case 5). (**A**) A 15-year-old boy presented with an asymptomatic, pink 5 × 6 mm papule on the lower left leg, which had been enlarging over the past few weeks. The clinical image shows a dome-shaped papule with smooth borders, while the dermoscopic view reveals symmetrical light brown pigmentation with regularly distributed dark dots. (**B**) Hematoxylin and eosin (HE) staining shows a symmetrical and well-circumscribed melanocytic proliferation with exophytic protrusion into the epidermis. (**C**) HMB45 staining highlights positive junctional melanocytes, negative dermal components, and central loss of HMB45 expression. (**D**) Melan A staining reveals positivity in spindle-shaped melanocytes with smaller nests observed deeper in the dermis. (**E**) Ki-67 staining demonstrates numerous positive cells, indicating high proliferative activity. (**F**) p16 staining shows a heterogeneous expression pattern with partial positivity and predominantly negative melanocytes. Molecular findings for Case 5 are summarized in Figure 6.

**Figure 4:**
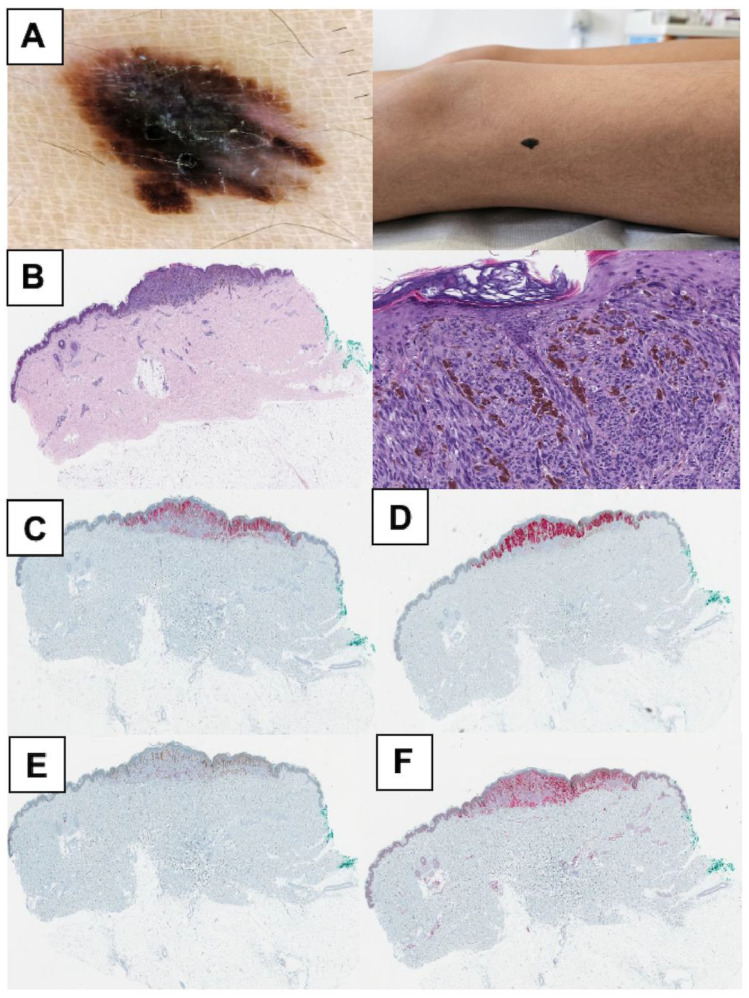
Summary of clinical, histological, and immunohistochemical findings in an atypical Spitz tumor patient (Case 6). (**A**) An 11-year-old girl presented with an asymptomatic, darkly pigmented 15 × 12 mm macula on the lateral left upper thigh, which was growing over a few months. The clinical image shows an irregularly shaped macule with uneven pigmentation and indistinct borders. The dermoscopic view reveals an asymmetric pattern with multiple shades of brown and black, irregular borders, and radial streaks of pigmentation extending peripherally. (**B**) Hematoxylin and eosin (HE) staining shows a heterogeneous lesion with areas of giant cells and marked atypia. (**C**) HMB45 staining demonstrates a stratification pattern towards the deeper parts of the lesion. (**D**) Melan A staining shows clear depiction of melanocytes with symmetrical architecture, predominantly found suprabasally underneath areas of parakeratosis. (**E**) Ki-67 staining shows single positive cells in the dermis. (**F**) p16 staining shows no evidence of complete antigen loss. Molecular findings for Case 6 are summarized in Figure 6.

**Figure 5:**
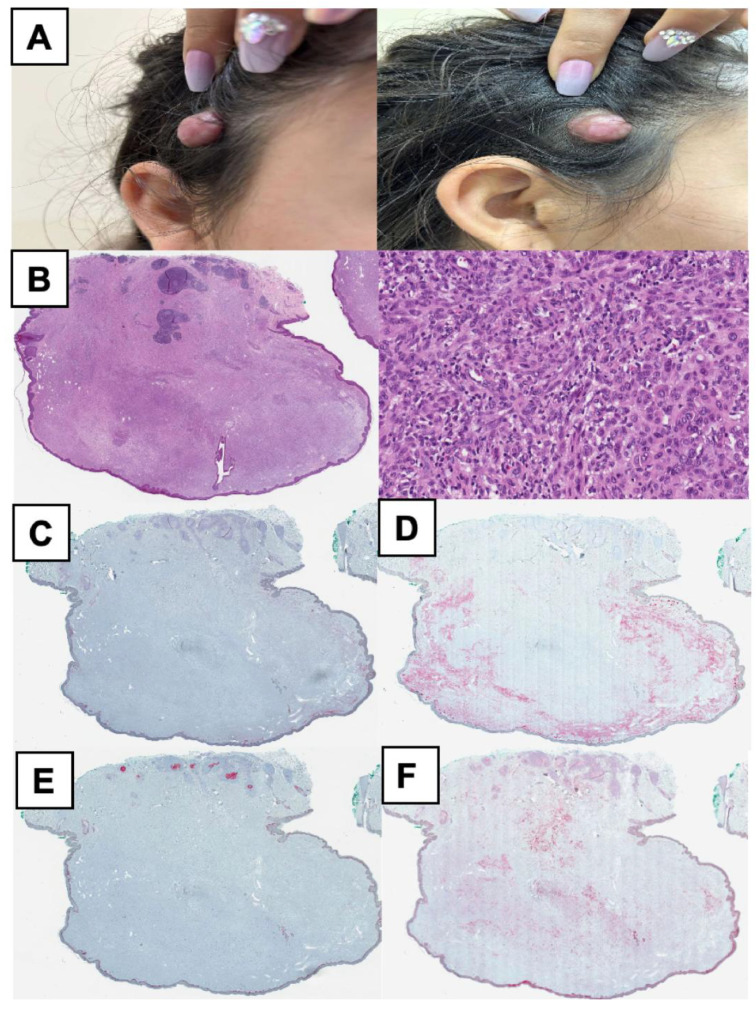
Clinical, histopathological, and immunohistochemical features of an atypical Spitz tumor (Case 4). (**A**) A 15-year-old female presented with a 30 × 30 mm nodule on the right temporal area. Present since birth, the lesion had gradually increased in size over the years. The clinical image shows a large, smooth, dome-shaped, skin-colored nodule with a lobulated appearance. The lesion is well-circumscribed and protrudes above the surface of the skin. (**B**) Hematoxylin and eosin (HE) staining reveals a large, highly cellular structure composed of various spindle-shaped and epithelioid cells, intermixed with multinucleated giant cells. The epithelioid regions exhibit significant nuclear pleomorphism and hyperchromasia, accompanied by a patchy pseudolymphomatous infiltrate at the lesion’s periphery. (**C**) HMB-45 shows positive staining predominantly in melanocytes located near the epidermis. (**D**) In the Melan A stain, about 30% of the tumor cells, primarily at the periphery, display positivity. (**E**) Ki-67 does not indicate increased proliferation, suggesting low proliferative activity within the lesion. (**F**) p16 staining reveals a substantial loss of antigen expression, with positivity observed in fewer than 10% of the tumor cells. Molecular findings for Case 4 are summarized in Figure 6.

The SM group included only one sample, further restricting the generalizability of these findings. Figure 3, Figure 4 and Figure 5 offer supplementary insights by presenting histological and IHC slides and the initial clinical presentation.

### 3.3. Next-Generation Sequencing Analysis

**Figure 6:**
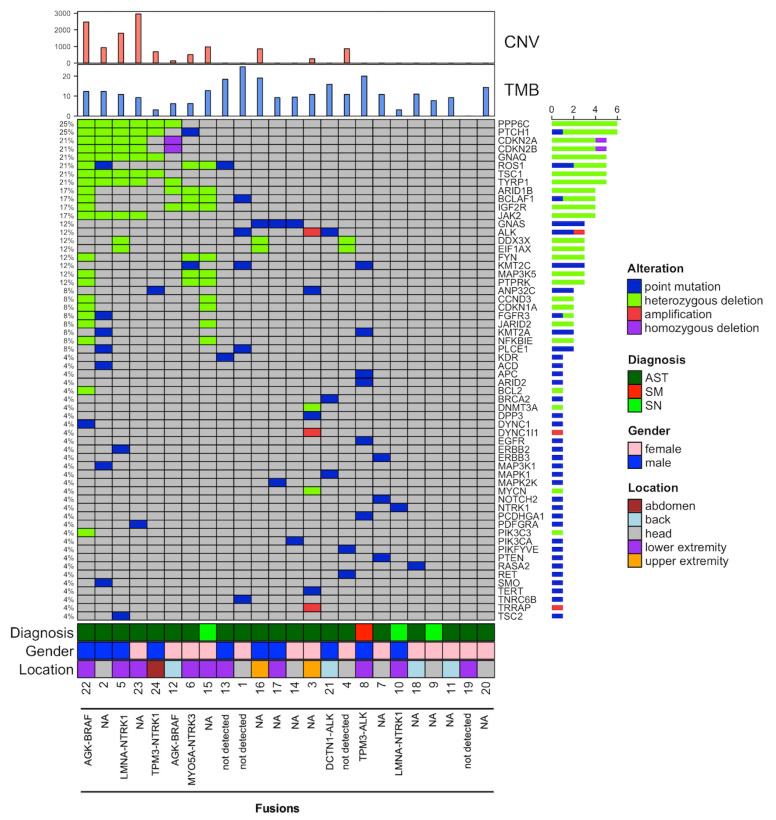
Genetic and clinical characteristics in Spitz tumors: this oncoprint displays the genetic alterations and clinical characteristics of 24 Spitz tumors. Each row represents a patient, with genetic alterations across various genes, including point mutations, deletions, amplifications, and fusion events, when available. If fusion analysis was not performed for a patient, “NA” is indicated. Diagnostic groups include atypical Spitz tumor (AST, dark green), Spitz nevi (SN, light green), and Spitz melanoma (SM, red). Additional features, such as tumor mutational burden (TMB) and copy number variation (CNV), are also shown.

**Figure 7:**
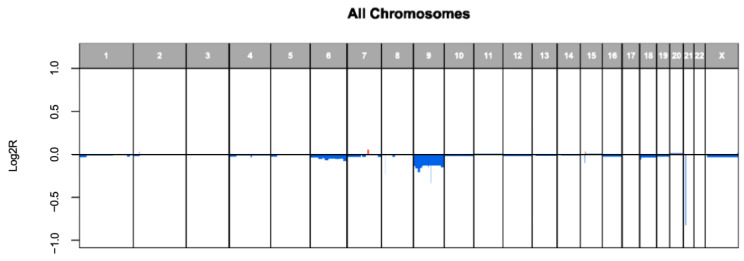
Copy number variations (CNVs) in the cohort analyzed using MelArray. CNVs detected in the cohort are displayed, restricted to chromosomes where CNVs were identified. Chromosomal segments are represented as log2R values, with positive (red) deviations indicating gains and negative (blue) deviations indicating deletions. This segmented representation visualizes the genomic alterations detected in the analyzed Spitz tumors.

Alterations were detected in 20 (83%) of the analyzed STs with a total of 132 alterations. Heterozygous deletions were the most common, accounting for 79 (59.8%) of all genetic alterations, followed by 48 point mutations (36.4%). Amplifications and homozygous deletions were less common, with two amplifications (2.3%) and three homozygous deletions (1.5%) identified. Testing for gene fusions was performed in 12/24 cases (50%), with fusion detected in 8 cases (66.7%) (Figure 6 and Figure 7, Table 3).

### 3.4. Subgroup Analysis

In the AST subgroup (*n* = 20), 3 tumors (15%) had no genetic alterations detected, while the remaining 17 tumors (85%) showed a range of genetic changes. Heterozygous deletions were the most common, accounting for 69 alterations (60.5%), followed by 40 point mutations (35.1%). Amplifications (three cases, 2.6%) and homozygous deletions (two cases, 1.8%) were less frequent and were detected exclusively in this subgroup. The most frequently observed mutations in this subgroup were heterozygous deletions in PPP6C found in six patients (30%). Heterozygous deletions in the genes PTCH1, GNAQ, TSC1, and TYRP1 were also frequently observed, each identified in five patients (25%). One patient exhibited homozygous deletions in both CDKN2A and CDKN2B (each 5%). Amplifications in the ALK, DYNC1I1, and TRRAP genes were found in one tumor, which also showed a point mutation in TERT (each 5%). The mean TMB was 11.18 ± 5.40 mut/Mb, and the CNV analysis showed a mean of 574.80 ± 877.70. NGS-based fusion analysis was conducted in 11 (55%) patients. In five cases (45.5%), no fusions were found. The detected translocation-induced fusions were as follows: AGK-BRAF in two patients (18.1%) and MYO5A-NTRK3, LMNA-NTRK1, DCTN1-ALK, and TMP3-NTRK1 each in one patient (9.1%). NA indicates data not available.

In the SN subgroup (*n* = 3), one tumor showed no detectable genetic alterations in the MelArray panel, but a LMNA-NTRK1 fusion was identified. This tumor was the only one in the SN subgroup for which fusion testing was performed. One tumor had only a point mutation in NTRK1, while the remaining tumor showed 11 heterozygous deletions, including in ROS1, BCLAF1, and ARID1B, which were frequently altered in the entire cohort. No amplifications or homozygous deletions were found. The mean TMB in the SN subgroup was 7.83 ± 4.80 mut/Mb, and the CNV analysis showed a mean of 327.67 ± 567.54.

The SM (*n* = 1) exhibited a limited yet distinct pattern of genetic alterations. A total of six point mutations were detected, affecting the ARID2, EGFR, KMT2A, KMT2C, PCDHGA1, and APC genes. All but KMT2A and KMT2C were exclusive for this tumor. No other types of alterations, such as amplifications or deletions, were observed. The TMB was high with 20 mut/mb, but interestingly, no CNVs were identified. Additionally, a TMP3-ALK fusion was found in this tumor.

## 4. Discussion

In younger individuals, superficial spreading melanoma (SSM) and nodular melanoma (NM) are the most prevalent melanoma subtypes, frequently driven by BRAF mutations [29,30]. While most melanocytic lesions have well-characterized driver alterations [4,8,10,12,24,31], STs follow various genetic pathways and exhibit significant heterogeneity in their oncogenic drivers [8]. Unlike the relatively predictable genetic pattern seen in early melanoma development [32], STs exhibit a wide spectrum of lesions ranging from benign SN to clearly malignant SMs, with numerous atypical intermediate forms in between [8,16,33]. This heterogeneity poses challenges in establishing standardized diagnostic and prognostic criteria.

With our study, we assessed the diagnostic value of a melanoma-specific NGS panel combined with clinical, histological, and IHC analysis to improve the assessment of diagnostically challenging ST lesions. Our findings revealed that most ambiguous ST lesions displayed minor heterozygous deletions or single point mutations, rather than the damaging mutation profile characteristic of SMs [24]. The IHC analysis of PRAME, Melan A, and PHK markers (p16, HMB45, Ki-67) demonstrated higher scores in ASTs compared to SN. This integrated approach proved useful for distinguishing malignant cases from those likely to follow a benign course, aiding in clinical decision making for better patient management.

Histopathological and IHC examination remains the most widely used and accessible method for classifying STs alongside clinical features [8,34]. In particular, patient age is a crucial consideration, as older individuals are more likely to develop malignancy [8]. Consistent with the literature, our cohort exhibited a similar age distribution (2–46 years old, median 15), a slight female predominance, and a tendency for tumors to occur primarily on the lower extremities and head [3,35,36,37]. In most of the assessed cases, clinical, histopathological, and IHC evaluation was sufficient to classify them as benign SN (*n* = 147) or ASTs (*n* = 37). However, 24 lesions displayed features which were concerning for melanoma, such as an elevated Ki-67 proliferation index and/or partial or complete loss of p16 expression, prompting an investigation via NGS.

In a prospective study by Gassenmaier et al. [38], a diagnosis of 50% of AST cases was revised after molecular diagnostic assessment, underscoring its essential role in the classification of ambiguous ST lesions. However, the interpretation of the NGS results for Spitz neoplasms remains an active area of research, and a standardized NGS panel has yet to be established [8]. In our AST cohort, NGS analysis revealed primarily small, heterozygous deletions affecting genes such as PPP6C, PTCH1, CDKN2A, and CDKN2B. While IHC markers raised some concerns, NGS did not uncover impactful mutations in the key regulatory pathways governing senescence, apoptosis, or DNA repair. The TMB and CNV profiles aligned with the detected alterations and showed no indications of widespread genomic instability. These findings, collectively, supported the classification of 20/24 cases as ASTs, rather than melanomas. In contrast, the SMs were characterized by distinct mutations in ARID2, EGFR, and APC, along with a high TMB of 20 mut/mb. This profile reflects an increased malignancy risk, and an elevated likelihood of tumor spread, setting it apart from less aggressive ST variants. In this case, a SNLB was performed, which yielded a positive result, and the patient subsequently underwent adjuvant immunotherapy. The patient remains disease-free 28 months after the diagnosis. In one notable AST case, we identified a homozygous deletion of CDKN2A, a tumor suppressor linked to increased malignancy risk in STs [12,14]. This deletion co-occurred with CDKN2B loss, heterozygous deletions in ARID1B, IGF2R, PPP6C, and TYRP, and a low TMB. Although these alterations may enhance the survival in lymphatic environments, the absence of additional high-risk mutations (e.g., TERT-p, TP53, PTEN, ARID) suggests intact protective mechanisms, limiting the malignant potential. As a result, the lesion was classified as an AST rather than an SM. Previous studies have highlighted the significant role of TERT-p mutations in driving aggressive tumor behavior and poor prognosis across various cancers, including cutaneous melanoma [39]. In a study of 56 spitzoid lesions by Lee et al. [14], TERT-p mutations were found in all four cases exhibiting aggressive behavior and metastatic spread, while they were absent in the 52 cases with favorable outcomes, supporting its role as a discriminating marker. However, additional alterations, such as homozygous deletions in CDKN2A or BRAF mutations, co-occurred in selected cases. A BRAF mutation suggests that the lesion is more likely a conventional melanoma with spitzoid features rather than a true SM. In our analysis, no TERT-p mutations were found. However, one patient from the AST subgroup had a mutation in the TERT gene outside the promoter region, which was considered non transforming. Three lesions were classified as ASTs despite no alterations being detected. These likely represent low-grade ASTs with atypical IHC findings, closer to the benign end of the Spitz tumor spectrum. While the absence of detectable mutations supports a benign diagnosis, the possibility of undetected alterations outside the panel’s scope cannot be entirely excluded. Among the three SN tumors, minimal genetic alterations were observed, along with a low TMB and CNV, supporting the notion of a more stable genome in this subgroup. The alterations identified, including heterozygous deletions or isolated point mutations, appear to be non-pathogenic, suggesting a benign nature for these tumors. This genetic stability is consistent with the observed low IHC scores.

Spitzoid melanomas (e.g., BRAF mutated) were not included in our study. Previous research suggests that many lesions diagnosed as an SM or AST, that developed distant metastases and resulted in a fatal outcome, were actually conventional melanomas harboring BRAF or NRAS mutations, frequently accompanied by TERT-p alterations [16,20,24,40,41]. While our study did not directly assess this, we emphasize that the MelArray panel is designed to detect these key driver mutations. A possible disadvantage of the panel is its inability to detect gene fusions, which constitute a significant portion of the genetic landscape in STs [4,8,16]. In our study, additional NGS-based fusion analysis identified rearrangements involving ALK and NTRK1 in 8/12 cases, allowing these lesions to be classified within the ST family. All the observed fusions have been previously described in the literature [18,19,42]. Although kinase fusions are key drivers in the development of STs, their presence alone does not reliably predict biological behavior or malignant potential [8]. Testing for fusion events can aid in distinguishing STs from other melanocytic lesions with a similar morphology. However, in our cohort, the detected rearrangements did not provide additional information that influenced clinical decision making. Additionally, no HRAS mutation was identified in any tumor, suggesting that alternative genetic pathways may be more prevalent in our cohort.

The MelArray NGS panel helps to identify Spitz lesions at risk of malignant clinical behavior by detecting the key mutations associated with tumor progression. It serves as a practical tool to highlight critical lesions. In our cohort, the panel demonstrated its diagnostic reliability, particularly for ASTs, as no local recurrences, distant metastases, or tumor-related deaths were observed during the follow-up. Nevertheless, the small cohort size, especially the single SM case, limits the generalizability of the findings on its molecular profile and progression. As NGS panels like MelArray become more accessible, their broader use could enable earlier malignancy detection and improved risk assessment for ambiguous cases. Multicenter studies with larger cohorts and extended follow-ups are essential to validate these findings, refine the classification, and enhance the risk stratification for better clinical decision making.

## 5. Conclusions

Since the treatment and prognosis for STs can vary significantly, especially as most affected individuals are very young, a comprehensive diagnostic approach is essential. The intention should be to identify patients with a potentially malignant course at an early stage while avoiding overdiagnosis and unnecessary treatment in ST patients who generally have a favorable outlook. While STs exhibit a distinct mutational landscape compared to cutaneous melanomas, the MelArray panel reliably detects key alterations associated with melanomas, particularly those critical for evaluating the lesion’s biological behavior (e.g., TERT-p, ARID2, TP53, or CDKN2A) [1,8,16]. In addition, the assessment of the TMB and CNV helps estimate the genomic alteration load, as higher mutation and CNV levels are frequently linked to an increased malignancy risk [40,43,44]. We therefore regard the MelArray panel not as a tool to precisely map each Spitz lesion along the benign-to-malignant spectrum, but rather to identify lesions at risk of malignant transformation by targeting the critical mutations associated with tumor progression. This provides a practical and reliable approach for early malignancy detection and helps guide appropriate management strategies.

## Figures and Tables

**Table 1:** Scoring criteria for immunohistochemical markers.

HMB45 (*n* = 21)	p16 (*n* = 23)	Melan A (*n* = 23)	PRAME (*n* = 11)	Ki-67 (*n* = 20)
0: Gradient present	0: >50% positive cells	2: Positive	2: Positive	0: <2% positive cells
1: Gradient inconclusive	1: 11–50% positive cells	1: Weakly positive	1: Weakly positive	1: 2–5% positive cells
2: Gradient absent	2: 1–10% positive cells	0: Negative	0: Negative	2: 6–10% positive cells
	3: 0% positive cells			3: 11–20% positive cells
				4: >20% positive cells

**Table 2:** Patient demographics and clinical features. Abbreviations: Spitz nevi (SN), atypical Spitz tumor (AST), Spitz melanoma (SM), LE: lower extremities, UE: upper extremities, SNLB: sentinel lymph node biopsy.

Patients (*n* = 24)	
Diagnosis	SN: 3 (12.5%) AST: 20 (83.3%) SM: 1 (4.2%)
Gender	female: 14 (58%) male: 10 (42%)
Age (years)	median: 15 range: 2–46
Location	LE: 10 (42%) head: 7 (29%) back: 4 (17%) UE: 2 (8%) abdomen: 1 (4%)
Re-excision	yes: 18 (75%) no: 6 (25%)
Safety margin (cm)	0.5: 12 (67%) 1: 5 (28%) 2: 1 (5%)
SNLB	yes: 3 (12%) no: 21: (88%)
SNLB positive	yes: 1 (33%) no: 2 (67%)
Follow-up (*n* = 23, months)	median: 36 range: 6–48
Local recurrence Distant metastasis Death	0/23 (0%) 0/23 (0%) 0/23 (0%)

**Table 3:** Mean scores ± standard deviations for alterations detected by the MelArray panel across patient subgroups: Cohort, Spitz nevi (SN), atypical Spitz tumors (ASTs), and Spitz melanoma (SM). The table includes the number of patients (*n*), total mutation count, and mean mutation score ± standard deviation for each subgroup, with missing values indicated as “NA”.

Group	Not Detected	Total Count	Mean	*n*	Amplification	*n*	Heterozygous Deletion	*n*	Homozygous Deletion	*n*	Point Mutation
Cohort (*n* = 24)	4	132	5.50 ± 5.29	3	0.13 ± 0.61	79	3.21 ± 5.41	2	0.08 ± 0.40	48	2.00 ± 1.09
SN (*n* = 3)	1	12	4.00 ± 4.97	0	NA	11	3.67 ± 6.35	0	NA	1	0.33 ± 0.577
AST (*n* = 20)	3	114	5.85 ± 5.60	3	0.15 ± 0.67	69	3.45 ± 5.46	2	0.10 ± 0.45	40	2.00 ± 1.75
SM (*n* = 1)	0	6	6.00 ± NA	0	NA	0	NA	0	NA	6	6.00 ± NA

## Data Availability

The data supporting the findings of this study are not publicly available due to privacy and ethical restrictions. Access to anonymized data may be provided upon reasonable request to the corresponding author and with the approval of the local ethics committee, in compliance with applicable regulations.

## Supplementary Materials

The following supporting information can be downloaded at: https://www.mdpi.com/article/10.3390/cancers17071227/s1, Table S1: Gene list covered by the MelArray panel, including their corresponding transcript IDs.
