# Supplementary material for: A Retrospective Analysis of Ambiguous Spitz Tumors Using Next-Generation Sequencing
Source: Cancers (Basel). 2025 Apr 4;17(7):1227. doi: 10.3390/cancers17071227 (PMC11988030; doi:10.3390/cancers17071227)
Supplement: Supplementary file 1 [file cancers-17-01227-s001.zip › cancers-3533233-supplementary.pdf]

Supplementary materials

ETV6	***EZH2***	***FAM58A***	***FANCA***	***FBXW7***	***FGFR1***
full<br>NM_001987.4	full<br>NM_004456.2	full<br>NM_152274.1	full<br>NM_000135.2	full<br>NM_033632.3	full<br>NM_001174067.1
FGFR2	***FGFR3***	***FGFR4***	***FYN***	***GNA11***	***GNAI2***
full<br>NM_022970.3	full<br>NM_001163213.1	full<br>NM_213647.2	full<br>NM_002037.1	full<br>NM_002067.4	full<br>NM_002070.3
GNAQ	***GNAS***	***HERC2***	***HLA-A***	***HLA-B***	***HLA-C***
full<br>NM_002072.4	full<br>NM_080425.3	full<br>NM_004667.5	full<br>NM_001242758.1	full<br>NM_005514.7	full<br>NM_002117.5
HRAS	***IDH1***	***IGF2R***	***IQGAP1***	***ITGA5***	***JAK1***
full<br>NM_005343.2	full<br>NM_001282387.1	full<br>NM_000876.3	full<br>NM_003870.3	full<br>NM_002205.4	full<br>NM_002227.2
JAK2	***JARID2***	***KDR***	***KIT***	***KMT2A***	***KMT2B***
full<br>NM_004972.3	full<br>NM_004973.3	full<br>NM_002253.2	full<br>NM_000222.2	full<br>NM_001197104.1	full<br>NM_014727.2
KMT2C	***KMT2D***	***KNSTRN***	***KRAS***	***MAP2K1***	***MAP2K2***
full<br>NM_170606.1	full<br>NM_003482.3	full<br>NM_033286.3	full<br>NM_033360.3	full<br>NM_002755.3	full<br>NM_030662.3
MAP2K4	***MAP3K1***	***MAP3K2***	***MAP3K5***	***MAP3K8***	***MAP3K9***
full<br>NM_001281435.1	full<br>NM_005921.1	full<br>NM_006609.4	full<br>NM_005923.2	full<br>NM_005204.1	full<br>NM_033141.3
MAPK1	***MAPK3***	***MC1R***	***MET***	***MITF***	***MLH1***
full<br>NM_002745.4	full<br>NM_002746.2	full<br>NM_002386	full<br>NM_001127500.2	full<br>NM_000248.2	full<br>NM_000249.3
MLH3	***MTOR***	***MYCN***	***NF1***	***NFKBIE***	***NOTCH2***
full<br>NM_001040108.1	full<br>NM_004958.3	full<br>NM_005378.4	full<br>NM_000267.1	full<br>NM_004556.2	full<br>NM_024408.3
NRAS	***NTRK1***	***OCA2***	***PARP1***	***PCDHGA1***	***PDGFRA***
full<br>NM_002524.4	full<br>NM_002529.2	full<br>NM_000275.2	full<br>NM_001618.3	full<br>NM_018912.2	full<br>NM_006206.4
PIK3C2A	***PIK3C3***	***PIK3CA***	***PIK3CB***	***PIK3R1***	***PIK3R4***
full<br>NM_001321378.1	full<br>NM_002647.3	full<br>NM_006218.3	full<br>NM_006219.1	full<br>NM_181523.2	full<br>NM_014602.2
PIKFYVE	***PKD2***	***PLA2G6***	***PLCB1***	***PLCE1***	***PLEKHG4***
full<br>NM_015040.2	full<br>NM_000297.3	full<br>NM_003560.1	full<br>NM_015192.3	full<br>NM_016341.3	full<br>NM_001129727.2
PMEL	***PMS2***	***POLQ***	***POT1***	***PPARG***	***PPP2R2A***
full<br>NM_006928.2	full<br>NM_000535.5	full<br>NM_199420.3	full<br>NM_015450.2	full<br>NM_015869.4	full<br>NM_001177591.1
ABCB5	***ACD***	***ACVR1C***	***AKAP9***	***AKT1***	***AKT2***
full<br>NM_001163941.1	full<br>NM_001082486.1	full<br>NM_145259.2	full<br>NM_005751.1	full<br>NM_001014431.1	full<br>NM_001626.5
AKT3	***ALK***	***ANP32C***	***APC***	***ARID1A***	***ARID1B***
full<br>NM_005465.4	full<br>NM_004304.4	full<br>NM_012403.1	full<br>NM_000038.2	full<br>NM_006015.4	full<br>NM_001346813.1
ARID2	***ARID4B***	***ARID5A***	***ASIP***	***ASPM***	***ATM***
full<br>NM_152641.3	full<br>NM_016374.5	full<br>NM_001319085.1	full<br>NM_001672.1	full<br>NM_018136.4	full<br>NM_000051.3
AURKA	***AURKB***	***BAP1***	***BCL2***	***BCL2L12***	***BCLAF1***
full<br>NM_003600.2	full<br>NM_001284526.1	full<br>NM_004656.2	full<br>NM_000633.2	full<br>NM_138639.1	full<br>NM_014739.2
BRAF	***BRCA1***	***BRCA2***	***CBL***	***CCND1***	***CCND2***
full<br>NM_004333.1	full<br>NM_007300.3	full<br>NM_000059.3	full<br>NM_005188.3	full<br>NM_053056.2	full<br>NM_001759.3
CCND3	***CDC42***	***CDK4***	***CDK6***	***CDKN1A***	***CDKN1B***
full<br>NM_001760.4	full<br>NM_001791.2	full<br>NM_000075.3	full<br>NM_001259.6	full<br>NM_001291549.1	full<br>NM_004064.4
CDKN2A	***CDKN2C***	***CHD8***	***CTNNB1***	***CXCL1***	***CYP1B1***
full<br>NM_001195132.1	full<br>NM_001262.2	full<br>NM_020920.1	full<br>NM_001904.2	full<br>NM_001511.3	full<br>NM_000104.3
CYP7B1	***DCT***	***DCUN1D3***	***DDR2***	***DDX3X***	***DLG1***
full<br>NM_004820.4	full<br>NM_001129889.2	full<br>NM_173475.3	full<br>NM_006182.4	full<br>NM_001356.4	full<br>NM_004087.2
DNMT1	***DNMT3A***	***DNMT3B***	***DPP3***	***DYNC1I1***	***E2F1***
full<br>NM_001130823.2	full<br>NM_153759.2	full<br>NM_006892.3	full<br>NM_005700.1	full<br>NM_004411.4	full<br>NM_005225.2
EGFR	***EIF1AX***	***EIF4A1***	***EP300***	***ERBB2***	***ERBB3***
full<br>NM_005228.4	full<br>NM_001412.3	full<br>NM_001416.3	full<br>NM_001429.3	full<br>NM_004448.3	full<br>NM_001982.3
PPP2R2B	***PPP2R5C***	***PPP3CA***	***PPP6C***	***PRAME***	***PRKAR1A***
full<br>NM_181675.3	full<br>NM_178586.1	full<br>NM_000944.4	full<br>NM_001123355.1	full<br>NM_206953.2	full<br>NM_212471.2
PRKCD	***PROS1***	***PTCH1***	***PTEN***	***PTPN11***	***PTPRF***
full<br>NM_212539.1	full<br>NM_001314077.1	full<br>NM_000264.3	full<br>NM_000314.4	full<br>NM_002834.3	full<br>NM_002840.4
PTPRJ	***PTPRK***	***RAC1***	***RAD51B***	***RAF1***	***RASA1***
full<br>NM_002843.2	full<br>NM_001291981.1	full<br>NM_018890.3	full<br>NM_001321821.1	full<br>NM_002880.3	full<br>NM_002890.2
RASA2	***RASA3***	***RB1***	***RET***	***RICTOR***	***ROS1***
full<br>NM_001303246.1	full<br>NM_007368.3	full<br>NM_000321.2	full<br>NM_020975.4	full<br>NM_001285439.1	full<br>NM_002944.2
RQCD1	***SETD2***	***SF3B1***	***SHOC2***	***SLC1A4***	***SLC45A2***
full<br>NM_005444	full<br>NM_014159.6	full<br>NM_012433.3	full<br>NM_007373.3	full<br>NM_003038.4	full<br>NM_016180.4
SMARCA4	***SMO***	***SOS1***	***SOS2***	***SPRED1***	***SPRED2***
full<br>NM_003072.1	full<br>NM_005631.4	full<br>NM_005633.3	full<br>NM_006939.3	full<br>NM_152594.2	full<br>NM_181784.2
SPRY4	***SRC***	***STK11***	***TAOK1***	***TAOK2***	***TERF2***
full<br>NM_030964.3	full<br>NM_005417.4	full<br>NM_000455.1	full<br>NM_020791.2	full<br>NM_004783.2	full<br>NM_005652.1
TERF2IP	***TERT***	***TNRC6B***	***TP53***	***TRRAP***	***TSC1***
full<br>NM_018975.3	full<br>NM_198253.2	full<br>NM_015088.2	full<br>NM_000546.5	full<br>NM_003496.1	full<br>NM_000368.4
TSC2	***TUSC3***	***TYR***	***TYRP1***		
full<br>NM_000548.4	full<br>NM_006765.2	full<br>NM_000372.4	full<br>NM_000550.2		

Table S1. Gene list covered by the MelArray panel, including their corresponding transcript IDs.
